# Ethanol/Naltrexone Interactions at the mu-Opioid Receptor. CLSM/FCS Study in Live Cells

**DOI:** 10.1371/journal.pone.0004008

**Published:** 2008-12-23

**Authors:** Vladana Vukojević, Yu Ming, Claudio D'Addario, Rudolf Rigler, Björn Johansson, Lars Terenius

**Affiliations:** 1 Department of Clinical Neuroscience, Karolinska Institutet, CMM L8:01, Stockholm, Sweden; 2 Department of Medical Biochemistry and Biophysics, Karolinska Institutet, Stockholm, Sweden; 3 Laboratory of Biomedical Optics, Swiss Federal Institute of Technology, Lausanne, Switzerland; Emory University, United States of America

## Abstract

**Background:**

Alcoholism is a widespread chronic disorder of complex aetiology with a significant negative impact on the individual and the society. Mechanisms of ethanol action are not sufficiently well understood at the molecular level and the pharmacotherapy of alcoholism is still in its infancy. Our study focuses at the cellular and molecular level on ethanol-induced effects that are mediated through the mu-opioid receptor (MOP) and on the effects of naltrexone, a well-known antagonist at MOP that is used clinically to prevent relapse in alcoholism.

**Methodology/Principal Findings:**

Advanced fluorescence imaging by Confocal Laser Scanning Microscopy (CLSM) and Fluorescence Correlation Spectroscopy (FCS) are used to study ethanol effects on MOP and plasma membrane lipid dynamics in live PC12 cells. We observed that relevant concentrations of ethanol (10–40 mM) alter MOP mobility and surface density, and affect the dynamics of plasma membrane lipids. Compared to the action of specific ligands at MOP, ethanol-induced effects show complex kinetics and point to a biphasic underlying mechanism. Pretreatment with naloxone or naltrexone considerably mitigates the effects of ethanol.

**Conclusions/Significance:**

We suggest that ethanol acts by affecting the sorting of MOP at the plasma membrane of PC12 cells. Naltrexone exerts opposite effects on MOP sorting at the plasma membrane, thereby countering the effects of ethanol. Our experimental findings give new insight on MOP-mediated ethanol action at the cellular and molecular level. We suggest a new hypothesis to explain the well established ethanol-induced increase in the activity of the endogenous opioid system.

## Introduction

The dramatic action of ethanol has puzzled scientists for many years, and the question whether ethanol evokes its effects in the brain by acting on lipids or proteins is the subject of a long-standing debate [1].

### Ethanol action on lipids or proteins

Early work focused on ethanol interactions with membrane lipids. According to the Meyer-Overton theory [2], ethanol, other organic solvents and anesthetics act by dissolving in the cell membrane, thus altering its fluidity and other biophysical parameters such as membrane volume, curvature and lipid phase transitions [3]. However, concentrations of ethanol that are relevant in alcohol use (10–40 mM) cause only minute increase in membrane fluidity, comparable with the effects that would be caused by temperature increase of less than one tenth of a degree Celsius [1]. Similar arguments, systematically reviewed in [1], have been raised against other hypotheses about ethanol acting on membrane lipids.

The introduction of electrophysiology shifted the focus to ethanol interactions with membrane proteins, primarily ligand-gated ion channels. Recent results with the GABA, NMDA and AMPA receptors suggest that ethanol can bind to a pocket in certain subunits, altering the sensitivity of the receptor to its ligand [4]. Again, concentrations of ethanol needed to produce changes in receptor function have been much higher than those commonly reached *in vivo* [1].

### The opioid response hypothesis

Interactions between alcohol and the CNS opioid signaling system are well established and documented in basic research and clinical practice [5]–[10] and the usefulness of the opioid-receptor antagonist naltrexone as an adjunct in the treatment of alcoholism has placed a spotlight on interactions between ethanol and opioid signaling. However, in spite of intensive research, mechanisms involving ethanol interaction with the opioid systems are not yet fully elucidated. The “opioid response” hypothesis asserts that ethanol increases the activity of the endogenous opioid system through the release of opioid peptides, which by interaction with the opioid receptors promote its reinforcing effects [11]–[13]. This hypothesis, supported by the demonstration that opioids stimulate the release of dopamine [3], has led to the assumption that medications blocking opioid activity may impede the reinforcing aspects of ethanol. However, this hypothesis was never proven to be correct and attempts to measure ethanol-induced increase in endogenous opioids levels have failed so far.

### The lipid-raft hypothesis of ethanol action

At all levels of organization of a living organism, signaling processes rely on a tight dynamic regulation and synchronization of signaling molecules and receptors as a critical factor in signal transduction. Emerging evidence indicates that membrane compartmentalization into lipid-enriched micro-domains, often termed lipid rafts plays an important role [14]. The diameter of lipid rafts varies from a few to several hundred nanometers, and they are enriched in specific lipids like cholesterol and sphingomyelin [15]. Certain proteins are enriched in rafts, others are embedded in the membrane surrounding the rafts, and some proteins can shuttle between raft and non-raft membrane compartments, depending on the functional status of the cell. Lipid rafts have been suggested to function as platforms connecting receptor complexes and their signaling pathways [14].

The notion that lipids in model and native membranes are structurally organized in micro-domains [16] brought about the idea that ethanol modifies local properties of lipid membranes by affecting the lipid dynamics [17] and/or structural organization. Recent results indicating that ethanol alters the lipopolysaccharide-induced redistribution of components of the Toll-like receptor 4 complex within the domain structure of the cell membrane [18], [19], have led Szabo et al. [20] to propose that ethanol influences the entry or exit of receptor to/from rafts at a lower concentration than needed for many other effects of ethanol. Plasma membrane structure, epitomized by lipid rafts, was also proposed to play a key role in ethanol-induced oxidative stress of the liver [21].

Our aim is to investigate cellular and molecular mechanisms of ethanol action at the level of the mu-opioid receptor (MOP) and the plasma membrane lipids. In addition, we study the molecular mechanisms of naltrexone action, a MOP antagonist used in alcoholism treatment. Quantification of molecular numbers and movement is achieved by FCS/CLSM (Fluorescence Correlation Spectroscopy/Confocal Laser Scanning Microscopy). This methodology enables us to study subtle changes in MOP/plasma membrane lipid dynamics in live cells, using ethanol concentrations that are relevant for its behavioral effects, at low, clearly not intoxicating (10 mM), intermediate (20 mM) and high concentrations (40 mM).

## Results

### Characterization of the cellular model

**Figure 1:**
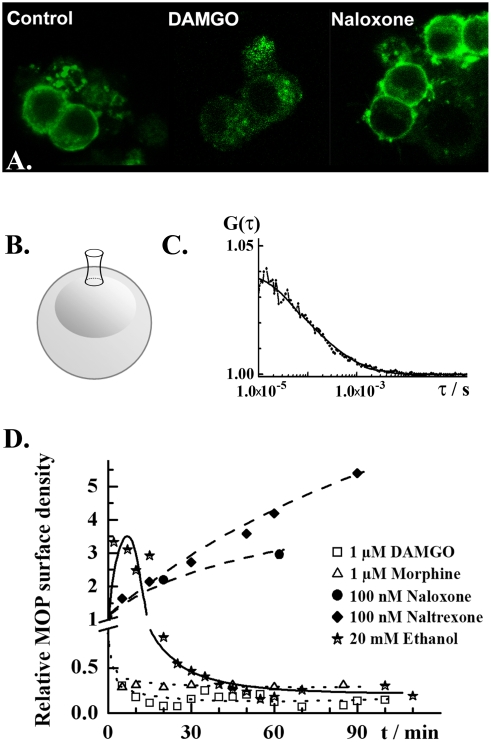
Effects of ethanol and opioid receptor agonists/antagonists on the MOP surface density in live PC12 cells. A. Confocal fluorescence images showing subcellular localization of MOP-EGFP (green) in PC12 cells under control conditions (left), upon 3 h treatment with DAMGO (1.0 µM; middle) or naloxone (100 nM; right). EGFP fluorescence was excited using the 488 nm line of the Ar laser. Fluorescence emitted in the range 505–540 nm was collected. B. Schematic drawing of a PC12 cell, showing the location of the observation volume element during FCS measurements. FCS measurements were always performed on the apical side of the plasma membrane. C. Typical autocorrelation curve for MOP-EGFP in not stimulated PC12 cells. FCS measurements were performed and analyzed as described in the *Materials and Methods* section. The dots give the experimental autocorelation curve; the smooth curve is a theoretical autocorrelation curve derived using a two-component model for free 2D-diffusion (eq. 3). Two fractions of MOP-EGFP were identified that could be distinguished by differences in lateral mobility, τ_D1_ = (250±150) µs and τ_D2_ = (2.5±1.5) ms. The majority of MOP-EGFP was characterized by fast mobility, *f*_1_ = (0.7±0.2). The amplitude of the autocorrelation curve is reciprocally proportional to the average number of MOP-EGFP molecules in the observation volume element (eq. 3). Autocorrelation curves are the basis for the calculation of relative changes in receptor surface densities as in the graph below. D. Relative changes in MOP-EGFP surface density under stimulation with selected drugs: ethanol (stars), naltrexone (diamonds), naloxone (dots), morphine (triangles) and DAMGO (squares). Selective ligands at MOP caused monotonous increase/decrease of MOP-EGFP surface density. Naloxone and naltrexone, acting as antagonists at MOP monotonously increased the MOP-EGFP surface density. The agonists DAMGO and morphine induced rapid internalization of MOP-EGFP, characterized by an internalization half-time *t*_1/2,agonists_ = 2.5 min. Ethanol induced an abrupt transient increase in MOP-EGFP surface density, followed by partial internalization of MOP-EGFP, with an apparent internalization half-time of *t*_1/2,ethanol_ = 25 min.

Cells derived from the rat pheochromocytoma (PC12) cell line were stably transformed to express the mu-opioid receptor (MOP) tagged with the Enhanced Green Fluorescent Protein (EGFP). The model is described and characterized in details in reference [22], where it was verified that the cloned construct MOP-EGFP exhibits properties similar to the native MOP–that the construct inserted in the plasma membrane, is sensitive and selective to its specific ligands and coupled to the cellular trafficking machinery (Fig. 1A).

MOP surface density and lateral mobility at the plasma membrane were measured using FCS, which is a quantitative method that relies on the analysis of fluorescence intensity fluctuations to determine molecular numbers and mobility. Theoretical background and essential information about the FCS methodology are given in the *Materials and Methods* section. FCS measurements were taken at the apical plasma membrane of PC12 cells (Fig. 1B). MOP surface density and mobility were determined by temporal autocorrelation analysis (Fig. 1C). For detailed explanation, see the *Materials and Methods* section.

We found that MOP expression levels differ strongly between cells, with local receptor densities ranging from 1–1000 molecules in the observation volume element. Assuming homogenous MOP distribution, the average number of receptors per cell was estimated to vary between 5×10^2^–5×10^5^. Only cells with local expression levels lower than 100 molecules per observation volume element were considered for further studies.

Two fractions of MOP were identified at the surface of PC12 cells that could be distinguished by differences in lateral mobility. The majority of MOP showed fast lateral mobility, τ_MOP,1_ = (250±150) µs. The slowly moving fraction, τ_MOP,2_ = (2.5±1.5) ms, dissipated upon cholesterol depletion and was enriched in detergent insoluble cellular extracts suggesting that this fraction corresponds to MOP associated with lipids and other components in detergent insoluble protein/lipid-rich micro-domains [22].

### Ethanol effects on MOP

**Figure 2:**
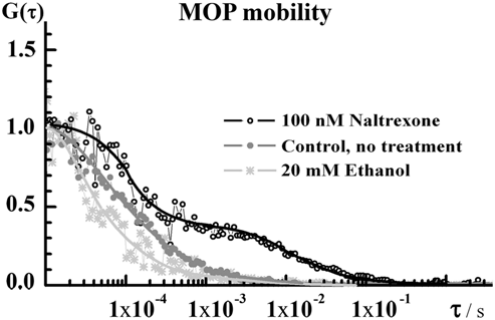
Normalized temporal autocorrelation curves showing the effect of ethanol and naltrexone on MOP-EGFP lateral mobility in the plasma membrane. The shift of the autocorrelation curve to shorter correlation times (stars) indicates that ethanol somewhat impels the lateral mobility of MOP-EGFP. In contrast, naltrexone markedly slowed down the lateral mobility of MOP-EGFP. The temporal autocorrelation curve (open circles) assumed a complex shape, indicating the formation of a slowly moving component.

In PC12 cells incubated with 20 or 40 mM ethanol, a transient increase in MOP surface density followed by partial MOP internalization was observed (Fig. 1D). Ethanol also increased the lateral mobility of MOP in the plasma membrane, as reflected by the shift of the autocorrelation curve towards shorter correlation times (Fig. 2, stars). In comparison to the effects of ethanol, specific ligands at MOP caused monotonous changes in receptor surface density. DAMGO and morphine caused a decrease in MOP surface density at similar rates, *k_int_* = (0.4±0.1) min^−1^, but to a different extent (Fig. 1D). Like ethanol, these agonists increased the lateral mobility of MOP (data not shown). The antagonists at MOP naloxone and naltrexone increased monotonously the surface density of MOP (Fig. 1D) and significantly reduced its lateral mobility (Fig. 2, open circles).

### Ethanol effects on plasma membrane lipids

**Figure 3:**
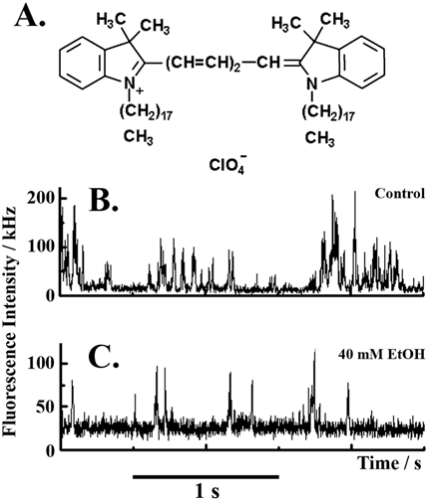
Dynamics of plasma membrane lipids under MOP stimulation with naltrexone and ethanol. A. Structural formula of the general lipid marker 1,1′-dioctadecyl-3,3,3′,3′-tetramethylindocarbocyanine perchlorate (DiIC_18_(5)) used as indicator of the plasma membrane lipid dynamics. DiIC_18_(5) was excited using the HeNe 633 nm laser. Fluorescence emitted in the range above 650 nm was collected. B. Fluorescence intensity fluctuations recorded in control cells. The corresponding temporal autocorrelation curve is shown in Fig 5A (black curve). C. Fluorescence intensity fluctuations showing the effect of 40 mM ethanol on the dynamics of plasma membrane lipids recorded after 15 min exposure to ethanol.

**Figure 4:**
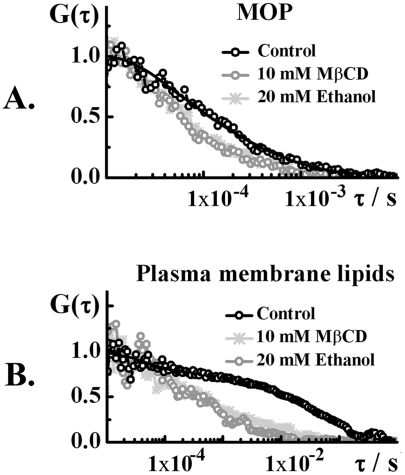
Effects of ethanol on the dynamics of MOP and plasma membrane lipids compared to the effects of cholesterol depletion by MβCD. Normalized autocorrelation curves showing changes in the dynamics of A. MOP and B. plasma membrane lipids, caused by 20 mM ethanol (dark gray) or 10 mM MβCD (light gray) as compared to the dynamics in control cells (black curve).

**Figure 5:**
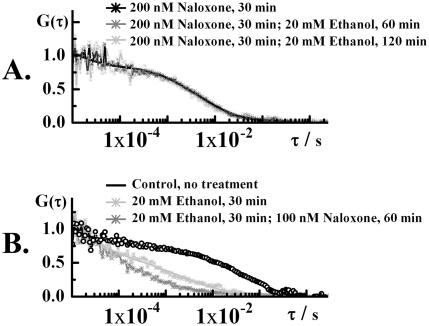
Modulation of ethanol-induced effects by naloxone. A. Normalized autocorrelation curves reflecting the dynamics of plasma membrane lipids in control cells (black curve), cells treated with 20 mM ethanol for 30 min (dark gray) and cells treated with 20 mM ethanol for 30 min, followed by treatment with 200 nM naloxone for 60 min (light gray). The characteristic decay time of the autocorrelation curves is decreased even after addition of naloxone. B. Normalized autocorrelation curves reflecting the dynamics of plasma membrane lipids in cells exposed to 200 nM naloxone for 30 min (black curve), cells treated with 200 nM naloxone for 30 min, followed by a 60 min exposure to 20 mM ethanol (dark gray) and cells treated with 200 nM naloxone for 30 min, followed by a 120 min exposure to 20 mM ethanol. The characteristic decay time of the autocorrelation curves remained unchanged, suggesting that the dynamics of MOP and plasma membrane lipids is largely unaffected. These findings suggest that effects of ethanol on the dynamics of MOP and the plasma membrane lipids are not easily reversed by naloxone or naltrexone. However, pre-treatment with these substances slows down the effects of ethanol.

The structural organization/dynamics of plasma membrane lipids was studied using the general fluorescent lipid marker DiIC_18_(5) (Fig. 3A). In untreated PC12 cells, the marker yielded complex fluorescence intensity bursts, consisting of alternating high/low-intensity spurts separated by prolonged intervals (about 0.7 s) of low-intensity fluorescence fluctuations (Fig. 3B). The intricate fluorescence pattern reflects complex dynamics/structural organization of plasma membrane lipids. Consequently, the corresponding autocorrelation curves were “stretched” and the characteristic decay times spanned over several orders of magnitude (Figs 4B and 5A).

After exposure to ethanol, marked changes in the lipid dynamics were observed (Figs 3C, 4B and 5A). As can be seen in Fig. 3, the large fluorescence intensity bursts (Fig. 3B) gradually disappeared and the fluctuation pattern changed its complex form to regular fluorescence intensity fluctuations (Fig. 3C). Correspondingly, the autocorrelation curve “shifted to the left”, showing shorter characteristic correlation times (Figs 4B and 5A).

### Ethanol effects on plasma membrane lipids are mediated through MOP

The effect of 20 mM ethanol on the mobility of MOP and the plasma membrane lipid dynamics were comparable to the effect of 10 mM methyl-β-cyclodextrin (MβCD), a compound known to extract cholesterol from the plasma membrane (Fig. 4). However, the underlying mechanisms seem to be different. As expected, the effects caused by MβCD did not depend on the presence of MOP (data not shown), whereas the effect of ethanol on plasma membrane lipids appeared to be enforced through MOP. In control cells, regular PC12 cells that express very low if any MOP, the direct effect of ethanol on the lipid dynamics was very slow and moderate changes could be observed only after prolonged, several hours' exposure (data not shown). This is in contrast with the modified, MOP expressing PC12 cells, where effects of ethanol on the plasma membrane lipid dynamics were readily observable shortly after exposure to ethanol. In addition, ethanol effects on the lipid dynamics could be modulated by specific ligands at MOP, as discussed in the following section.

### Naloxone and naltrexone modulate the effect of ethanol on MOP and plasma membrane lipid dynamics

The effects of naloxone or naltrexone on MOP-ethanol interactions were investigated in two paradigms. To study the preventive potential of naloxone/naltrexone, cells were pre-incubated with 100 nM naltrexone or 200 nM naloxone for 10–30 min and thereafter exposed to ethanol (10, 20 or 40 mM). Pre-exposure to the antagonists at MOP slowed-down the effects of ethanol (Fig. 5A).

The potential of naloxone/naltrexone to reverse the ethanol-induced effects was studied by exposing the cells to 20 mM ethanol for 30 min, followed by a treatment with naloxone/naltrexone. Effects caused by pre-incubation with ethanol could not be readily reversed by naloxone or naltrexone (Fig. 5B).

## Discussion

Due to its simple chemical structure, alcohol is often classified as a drug with a nonspecific primary site of action. The notion that ethanol interferes predominantly with membrane-associated processes has brought in focus plasma membrane lipids [1], [18]–[21] and plasma membrane proteins [1], [4], [23]. In early studies, these components were strictly delineated [1], although it has been suggested that ethanol acts both on proteins and their immediate surroundings, the so-called annular lipids, exerting its effects by disrupting the protein-lipid interactions [24]–[26]. Until recently such interactions were not easily accessible for study in live cells due to the lack of adequate experimental methodology. Current advances in molecular fluorescence imaging, in particular the FCS/CLSM methodology, now open the opportunity to study nondestructively molecular interactions in a restricted area of the plasma membrane (observation volume element about 400 nm in diameter), in real time and with single-molecule sensitivity [22].

We observed profound effects of ethanol on the MOP receptor surface density and mobility, as well as on the dynamics/organization of lipid constituents in the plasma membrane. Our findings, summarized in Table 1, show that effects of ethanol are markedly different from the effect of opioid-specific agonists and antagonists. Agonists at MOP cause fast receptor internalization with internalization half-times 2–5 min (Fig. 1D). Similarly to the effect of agonists, ethanol promotes MOP lateral mobility at the plasma membrane. Ethanol also induces MOP internalization; however the time course of ethanol-induced MOP internalization is complex and markedly different from the course of ligand-induced internalization (Fig. 1D). In contrast, antagonists at MOP appear to stall the receptor at the plasma membrane, cause monotonous increase in MOP surface density (Fig. 1D) and slow-down its lateral mobility (Fig. 2, open circles).

**Table 1:** A summary of ethanol effects at MOP and plasma membrane lipid dynamics compared to the effects of specific ligands.

Compound	MOP		Lipid dynamics	Probable cellular processes
	surface density	mobility		
DAMGO	Monotonous decrease	Increased	Fast	MOP internalization
Morphine	t_1/2_≈2–5 min			
Naltrexone	Monotonous increase	Decreased	Complex	MOP sorting*
Naloxone	t_1/2_≈20 min			
Ethanol	Transient increase followed by reduction	Increased	Fast	MOP sorting**/internalization

* Naltrexone and naloxone promote MOP association into larger molecular complexes.

** Ethanol dissipates the plasma membrane micro-domains, thereby releasing the “associated” form and rendering “free” MOP.

The transient increase in MOP surface density of (Fig. 1D) is especially interesting. FCS analysis reveals that relevant concentrations of ethanol induce a 3-fold increase in MOP surface density in 10 min. It was shown previously that MOP expression is not increased in PC12 cells exposed to ethanol [22], [27], suggesting that elevated MOP surface density is due to differences in trafficking and/or sorting of the existing pool of MOP. One possibility may be that MOP is replenished from the intracellular pool by faster recruitment of transporting vesicles to the plasma membrane. This may be a general protective mechanism that aims at “mending” the plasma membrane and restoring the properties that were affected by ethanol action. However, our preliminary studies on other types of opioid receptors suggest that this is not the main pathway. More likely, the apparent increase in MOP surface density reflects the redistribution of MOP at the plasma membrane. Oligomerization of opioid and other G-protein Coupled Receptors (GPCRs) [28]–[30] along with sorting in plasma membrane micro-domains [31] are known to take place at the plasma membrane. Dissociation of oligomers and/or release of multiple MOP molecules from micro-domains would be reflected in temporal autocorrelation analysis as an apparent increase in MOP surface density accompanied by enhanced lateral mobility, as we observed in our study. However, to prove unequivocally these mechanisms additional studies using brightness analysis [32] and Fluorescence Cross-Correlation Spectroscopy (FCCS) [22] are required.

Ethanol is known to affect cholesterol trafficking in CNS cells *in vitro* [33] and may exhibit cholesterol-reducing effects. Molecular dynamics simulations indicate that ethanol readily assembles in lipid bilayers (within 10 ns), enlarging the area per lipid and yielding a “softer” bilayer with enhanced lipid diffusion [34]. The effects of ethanol on plasma membrane lipid dynamics are comparable to the effect of the cholesterol depleting agent MβCD (Fig. 4). However, the effects of ethanol on the plasma membrane lipid dynamics appeared to be mediated through MOP and could be modulated by antagonists at MOP (Table 2). This finding is difficult to explain by the general mechanisms mentioned above.

**Table 2:** Naloxone and naltrexone modulate the effect of ethanol on the plasma membrane lipid dynamics.

Pre-treatment (time)		Ethanol/mM	Full effect of ethanol visible after
Naloxone/nM	Naltrexone/nM		
-	-	20	30 min
	100 (10 min)	40	50 min
200 (30 min)		20	>120 min

Based on the presented results, the following biphasic mechanism of ethanol action can be envisaged for MOP-mediated effects in PC12 cells. Ethanol dissipates the plasma membrane micro-domains, thereby releasing the “associated” form and rendering “free” MOP. This is reflected in FCS measurements as increase in MOP surface density, enhanced MOP mobility and intensified plasma membrane lipid dynamics. Increased surface density of “free” MOP potentiates the endogenous opioid signaling and activates the cellular internalization machinery, secondarily causing the sharp reduction in MOP surface density. Antagonists at MOP seem to work in the opposite direction–naltrexone and naloxone promote MOP association into larger molecular complexes, thereby countering and slowing down the action of ethanol.

### A new hypothesis on MOP-ethanol interaction

It is widely assumed that ethanol increases the activity of the endogenous opioid system through the release of opioid peptides and that anticraving/antihedonic effects of naltrexone (Revia®), used clinically to prevent relapse in alcoholism are achieved through antagonizing the effect of opioid peptides released by ethanol that are acting at the MOP receptor. However, attempts to measure ethanol-induced increase in endogenous opioids levels have failed so far. In addition, animal model studies showed that MOP knockout mice do not self-administer alcohol [35] and that neither null-mutation of preproenkephalin, nor homozygous knockout of proopiomelanocortin (the precursor of β-endorphin) affects the voluntary intake of ethanol in mice [36]–[38]. Thus, modification of β-endorphin or enkephalin levels, which are the endogenous peptide ligands at MOP does not affect the preference of ethanol intake in mice whereas the elimination of MOP does.

Our findings enable us to **speculate** on an alternative molecular mechanism for the well established ethanol-induced opioid surfeit. We suggest that ethanol affects MOP-mediated signaling by changing the distribution of MOP at the plasma membrane. It is possible that transient increase in “free” MOP, rather than the release of endogenous endorphins increases the activity of the endogenous opioid system. The subsequent reduction in MOP surface density (Fig. 1D) might explain the unpleasant feelings in the aftermath of exposure to ethanol and the finding that MOP density is reduced in certain brain areas after chronic ethanol intake [39]. In line with this reasoning, antagonists at MOP might counteract the effect of ethanol by sorting MOP to the “aggregated” state, thereby slowing down MOP redistribution, rather than by antagonizing the effect of endogenous opioid ligands.

It is important to underline that our results apply for the cellular model studied and the link between physiological actions of ethanol and the clinical effects of opioid antagonists needs still to be established.

### Summary

An important aspect of our study is the possibility to monitor local changes in protein and lipid dynamics and observe how it is affected by a non-specific substance like ethanol. The possibility to probe the lipid micro-environment around plasma membrane bound receptors [22], [40] may open a way to study important aspects of the role of lipids in cell surface receptor activation, an issue that is particularly relevant for the mechanism of action of pharmacological substances like general anesthetics that have the potential to perturb the lipid matrix and in this way may induce receptor mediated signaling cascades [41].

The experimental results presented in this study give new insight into the cellular and molecular mechanism of MOP-mediated ethanol action in the PC12 cellular model. Ethanol affects MOP mobility and the plasma membrane lipid dynamics, hinting at MOP release from micro-domains and redistribution at the plasma membrane. Naloxone and naltrexone apparently promote MOP association into larger molecular complexes and markedly slow down the effects of ethanol.

## Materials and Methods

### Confocal Laser Scanning Microscopy and Fluorescence Correlation Spectroscopy (CLSM/FCS)

Quantitative imaging was achieved by integrating Fluorescence Correlation Spectroscopy with Confocal Laser Scanning Microscopy (FCS/CLSM). Amalgamation of these methods yielded a hybrid instrument for quantitative imaging with the capacity to investigate dynamic processes in live cells in real time with single-molecule sensitivity.

### Instrumental setup

FCS/CLSM measurements were performed on a uniquely modified ConfoCor3 instrument (Carl Zeiss, Jena, Germany) consisting of an inverted microscope for transmitted light and epifluorescence (Axiovert 200 M); a VIS-laser module comprising the Ar/ArKr (458, 477, 488 and 514 nm), HeNe 543 nm and HeNe 633 nm lasers; scanning module LSM 510 META modified to enable detection using silicon avalanche photodiodes (SPCM-AQR-1X; PerkinElmer, USA) and an FCS module with three detection channels. Images were recorded at a 512×512 pixel resolution. The C-Apochromat 40×/1.2 W UV-VIS-IR objective was used throughout.

For imaging of live cells, a cell cultivation system consisting of a heated microscope stage (Heating insert P), incubator box (Incubator S), atmosphere-controlling device (CTI-Controller 3700) equipped with a humidifier and a temperature controlling device (Tempcontrol 37-2 digital) was applied. The CTI-Controller 3700 supplies the cultivation chamber with a heated mixture of CO_2_/air. The CO_2_ concentration and the temperature were continuously monitored and regulated by a digital feedback control algorithm, enabling a regulated dispersion of CO_2_ into the air stream and steady heating.

### Theoretical background on FCS

**Figure 6:**
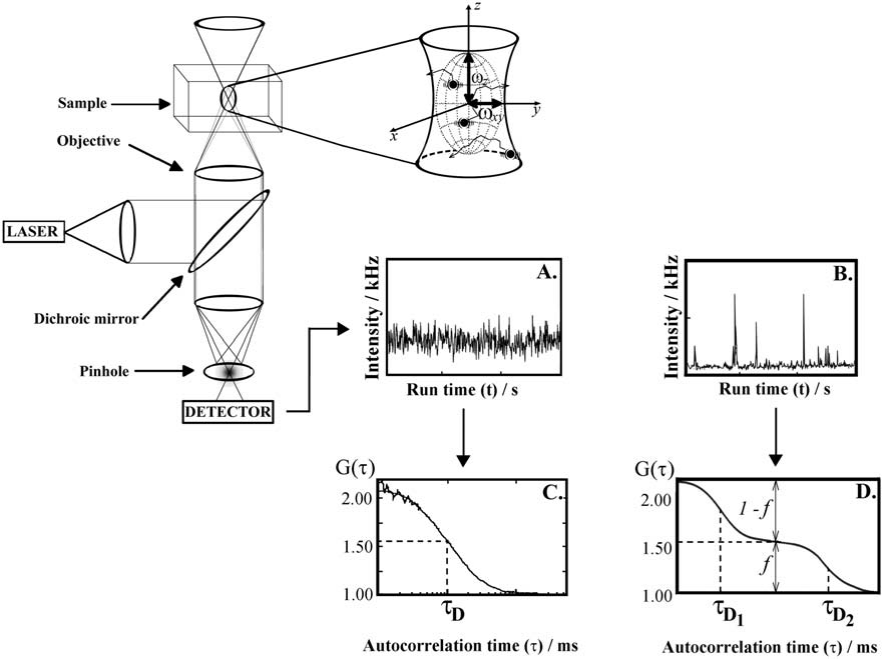
Schematic presentation of the instrumentation for FCS/CLSM. To induce fluorescence, the sample is illuminated by incident laser light. The irradiating laser beam is reflected by a dichroic mirror and sharply focused by the objective to form a diffraction limited volume element. A confocal aperture, set in the image plane to reject the out-of-focus light, further reduces the volume from which fluorescence is detected. This is crucial for providing an elliptical observation volume element and enabling submicrometer resolution and quantitative analysis. Following the absorption of energy, fluorescent molecules lose energy through photon emission. Light emitted by fluorescing molecules passing through the confocal volume element (magnified in the insert) is separated from the exciting radiation and the scattered light by a dichroic mirror and barrier filter, and transmitted to the detector. The number of pulses originating from the detected photons, recorded during a specific time interval, corresponds to the measured light intensity. Examples of fluorescence intensity fluctuations recorded in systems with one component undergoing A. free three-dimensional diffusion or B. free three-dimensional diffusion with binding to a surface. Corresponding autocorrelation curves are shown in C and D, respectively. C. Autocorrelation curve G(τ) fitted using equation (2a). The average number of molecules in the observation volume element is determined from the amplitude of the autocorrelation function (1/*N* = 0.94), and average residence time τ*_D_* from the inflection point. D. Autocorrelation curve G(τ) fitted using equation (2c). The complex shape of the autocorrelation curve indicates that two components with different diffusion times, τ*_D1_* and τ*_D2_*, are present. The average number of particles in the observation volume element is determined from the amplitude of the autocorrelation curve (1/*N* = 0.8). The ratio of the free fraction *versus* the bound is given by the ratio of the relative amplitudes (1−*f* )/*f*.

FCS is a physical method that relies on the measurement and analysis of fluorescence intensity fluctuations to characterize quantitatively the investigated system and extract information about the dynamics of processes leading to fluorescence intensity fluctuations [42]–[47]. To measure fluorescence intensity fluctuations in a diffraction limited volume element, the optical setup of a confocal microscope is used (a schematic presentation is given in Fig. 6). In the microscope, incident laser light is reflected by a dichroic mirror and sharply focused by the objective to form a diffraction limited volume element. A confocal aperture is set in the image plane to reject the out-of-focus light and enhance further the signal-to-noise ratio. The pinhole also reduces the volume from which fluorescence is detected, providing an elliptical detection volume element (magnified in the insert) with submicrometer resolution in two lateral directions–the diffraction limited detection volume element is typically smaller than 0.5 µm×0.5 µm×2 µm. Light emitted by fluorescing molecules passing through the observation volume element or undergoing transformations that lead to fluorescence emmision/loss is separated from the exciting radiation and the scattered light by the dichroic mirror and a barrier filter, transmitted to the detector, recorded in real time (Fig. 6A, B) and analyzed using statistical methods for fluctuation analysis. Most often, fluorescence intensity fluctuations are analyzed using temporal autocorrelation analysis (Fig. 6C, D), as done in this study, but other approaches can also be applied [45].

In temporal autocorrelation analysis the normalized autocorrelation function *G*(*τ*) is first derived. *G*(*τ*) gives the correlation between the deviation of fluorescence intensity, measured at a certain time *t*, ∂*I*(*t*) = *I*(*t*)−〈*I*(*t*)〉, and its intensity measured at a later time *t*+*τ*, ∂*I*(*t*+*τ*) = *I*(*t*+*τ*)−〈*I*(*t*)〉, from the average fluorescence intensity 〈*I*(*t*)〉:
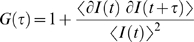
(1)


The normalized autocorrelation function *G*(*τ*) is then plotted for different autocorrelation times *τ*, yielding the experimental autocorrelation curve (Fig. 6C, D). In molecular systems undergoing stochastic fluctuations, one would observe random variations of *G*(*τ*) around the value *G*(*τ*) = 1. For processes that are not random, one typically observes a maximal limiting value of *G*(*τ*) as *τ*→0, decreasing to the value of *G*(*τ*) = 1 at long times, indicating that correlation between the initial and the current property value has been lost (Fig. 6C, D). In a simple system with one component and a single characteristic fluctuation frequency, the autocorrelation curve has a simple sigmoid form (Fig. 6C). The amplitude of the autocorrelation function *G*(*τ*) as *τ*→0, is inversely proportional to the absolute concentration of the fluorescing molecules, whereas the range of τ-values over which *G*(*τ*) changes rapidly as a function of τ gives the time scale at which the fluorescence intensity fluctuations occur. In a system with multiple components, for example two components that differ in their diffusion properties, the autocorrelation function assumes a more complex shape (Fig. 6D). The amplitude of the autocorrelation function as *τ*→0 is inversely proportional to the average number of all fluorescent particles in the observation volume element, multiple inflection points indicate that fluctuations in the signal occur at two time scales (τ_D1_ and τ_D2_) and the relative amounts of the two components can be estimated from the amplitudes 1−*f* and *f*.

In real experiments, the autocorrelation curves are fitted numerically using theoretical autocorrelation functions. To derive an appropriate autocorrelation function it is very important to take notice of all processes that may lead to fluctuations in the fluorescence signal, like diffusion, active transport, chemical reactions, structural transformation, photophysical processes *etc.* because all processes leading to statistical fluctuations in the fluorescence signal will induce a characteristic decay time in the autocorrelation curve [45]–[47]. For example, if the passage of fluorescent particles is governed solely by diffusion, the experimental autocorrelation curve (Fig. 6C) can be fitted by an autocorrelation function describing free three-dimensional diffusion of one component:
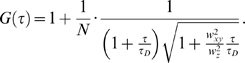
(2a)In equation (2a), *N* is the average number of fluorescent molecules in the observation volume element; *w_xy_* and *w_z_* give the 1/e^2^ radius of the observation volume element in the radial and axial direction, respectively; *τ_D_* is the average time a fluorescent particle stays in the observation volume element. In FCS terminology, *τ_D_* is called the lateral diffusion time and is directly related to the size of the observation volume element and the diffusion coefficient (D) of the fluorescent particles:
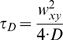
(2b)


If the example given in Fig. 6D represents ligand binding to plasma membrane associated receptors, the corresponding theoretical autocorrelation function would be:
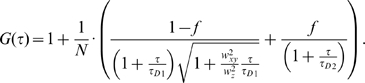
(2c)In equation (2c) *N* is the average number of ligand molecules in the observation volume element; the first term in the parenthesis describes free three-dimensional diffusion of unbound ligand molecules; the second term describes two-dimensional diffusion of the membrane-associated ligand-receptor complexes; *τ_D1_* and *τ_D2_* are the lateral diffusion times for the unbound and bound ligand, respectively. The relative amount of unbound *versus* bound ligand molecules is given by the ratio of (1−*f* ) over *f*.

### FCS data acquisition and analysis

Fluorescence intensity fluctuations were recorded in arrays of 10–30 consecutive measurements, each measurement lasting 5–10 s. Averaged curves were analyzed using the FCS/CLSM running software for online data analysis, or exported and fitted offline using the IGOR Pro 5 data analysis software (WaveMetrics, Inc. Portland, USA). In either case, the nonlinear least-square fitting of the autocorrelation curve was performed using the Levenberg-Marquardt algorithm.

The experimental autocorrelation curves were fitted using models for free two-dimensional diffusion:
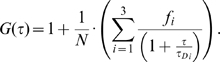
(3)Quality of the fitting was evaluated by visual inspection and by residuals analysis. The model with the smallest number of variables for which good fitting was achieved was selected as representative.

### Cell culture

PC12 cells were obtained from ATCC through LGC Promochem (Borås, Sweden). For cloning and multiplication purposes, the cells were cultured in collagen-coated flasks in RPMI 1640 medium supplemented with 5% fetal bovine serum, 10% heat-inactivated horse serum, 100 U/mL penicillin and 100 µg/mL streptomycin (all from Invitrogen, Sweden). The cells were maintained at 37°C in a humidified 5% CO_2_ incubator. The medium was replaced every 2–3 days. For CLSM and FCS experiments, the cells were plated on 8-well chambered coverslips (Nalge Nunc International, USA) and grown in phenol-red free RPMI medium supplemented with 10% horse serum, 5% fetal bovine serum, penicillin (100 units/mL) and streptomycin (100 µg/mL) in humidified 5% CO_2_ atmosphere at 37°C. Average cell density at plating was about 1×10^5^ cells/cm^2^, in 300 µL medium. The cells were subjected to CLSM/FCS analysis 2 to 3 days after plating in the chambered coverslips.

### Fluorescent staining of plasma membrane lipids

Lipophilic 1,1′-dioctadecyl-3,3,3′,3′-tetramethylindocarbocyanine perchlorate dye (DiIC_18_(5); Vybrant Cell-Labeling Solution V-22887, Molecular Probes/Invitrogen Labeling & Detection, Eugene, USA) was used as a general membrane stain [48], [49]. Cells were incubated with the labeling solution diluted with the culture medium (1∶200) for 7–10 min at 37°C. Shorter incubation times (2 min and 5 min) were also tested, but did not yield uniform staining of the cellular plasma membrane. Excess of the dye was removed by washing three times with the cell culture medium.

### Cholesterol depletion

A stock solution of 100 mM methyl-β-cyclodextrin (MβCD; Sigma-Aldrich, St. Louis, USA) was prepared in phosphate-buffered saline and stored at 4°C. Prior to the measurements, the stock solution was diluted with the culture medium to yield a 10 mM solution. We incubated the cells with 10 mM MβCD for 30 min at 37°C, a procedure that was shown not to be toxic for PC12 cells [50]; washed three times with the culture medium and stained with DiIC_18_(5) as described above. We did not observe any effect of the applied treatment on cellular viability during the observation time (several hours).
